# Tonic immobility behaviour does not differ between fire salamander larvae from ponds and streams

**DOI:** 10.1002/ece3.11211

**Published:** 2024-04-01

**Authors:** Laura Schulte, Barbara A. Caspers

**Affiliations:** ^1^ Department of Behavioural Ecology Bielefeld University Bielefeld Germany; ^2^ Joint Institute for Individualisation in a Changing Environment (JICE) University of Münster and Bielefeld University Bielefeld Germany

**Keywords:** amphibia, caudata, death‐feigning, defence behaviour, *Salamandra salamandra*, thanatosis

## Abstract

Tonic immobility is an antipredator defence in which the prey animal remains motionless after physical contact with the predator, pretending to be dead. This behaviour has been observed among a variety of taxa but has received only little attention in amphibian larvae. During our field studies with fire salamander larvae, we observed that larvae from different habitats display tonic immobility after handling. In our study site, we find larvae in pond and stream habitats, that differ in several aspects such as their stress response and their risk‐taking behaviour, likely caused by the very different habitat conditions. We measured the time that the tonic immobility behaviour was displayed but found no difference between larvae from the two habitat types. Likewise, we also found no correlation between the size of the larvae and the duration of displaying the behaviour. In conclusion, we found that fire salamander larvae show tonic immobility, but found no evidence that the different habitat conditions influence the tonic immobility behaviour.

## Graphical abstract

We showed that fire salamander larvae can display tonic immobility behaviour when being handled. In a genetically diverging population where larvae occur in pond and in stream habitats, we found no evidence for an effect of the larval habitat on the tonic immobility behaviour.

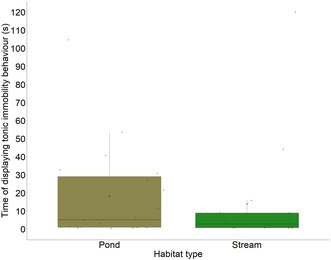

## 1 INTRODUCTION

Tonic immobility is an antipredator behaviour which is well known from several species (e.g., Damas‐Moreira, 2021; Konishi et al., 2020; Li et al., 2019; Sazima, 1974). It describes an innate and reversable reflex in which the individual remains motionless in a distinct posture pretending to be dead (Baškiera & Gvoždík, 2021; Páez et al., 2023; Toledo et al., 2010). Many predators do not consume dead prey and thus this behaviour is supposed to be an antipredator behaviour, increasing survival (Sazima, 1974; Teles et al., 2017). Tonic immobility often occurs after prey and predator are in physical contact and thus it is believed to be the last attempt of the prey to survive (Gallup, 1977; Humphreys & Ruxton, 2018, but see Damas‐Moreira, 2021, for an example of spontaneous tonic immobility). Interestingly, in the Central American cichlid (*Parachromis friedrichsthalii*), tonic immobility has also been described as a predatory behaviour, which individuals display to mimic a rotten fish to attract prey that scavenge on dead bodies (Tobler, 2005). Tonic immobility is also widespread in amphibians but has mostly been studied in anurans and the post‐metamorphic stage (e.g., Passos et al., 2017; Teles et al., 2017), whereas knowledge about the use of tonic immobility at the larval stage, especially in Caudata is lacking. However, Brodie et al. (1974) found one larva of *Paramesotriton hongkongensis* that started undergoing metamorphosis showing a defensive posture as well as hatchlings of *Aneides aeneus* and *Plethodon nettingi hubrichti*. While studying individual differences in larval fire salamanders (*Salamandra salamandra*), we observed larvae performing tonic immobility. Here, we describe the behaviour, and we tested, whether this behaviour is influenced by the larval habitat type, i.e., larvae that were found in ponds or streams.

Fire salamanders are widespread throughout Europe and they inhabit preferably deciduous forests with first order streams for larval deposition (Thiesmeier, 2004). Sometimes larvae are not only deposited into streams but also into ponds (Hendrix et al., 2017; Reinhardt, 2014; Steinfartz et al., 2007; Weitere et al., 2004). Interestingly, fire salamanders in the Kottenforst near Bonn (Germany) can be genetically distinguished according to the larval habitat type, pond or stream. Additionally, the larvae from the two habitat types show differences in their morphology (Schulte, 2008), physiology (Schulte et al., 2023) and behaviour (Hahn et al., 2023; Oswald et al., 2020).

Ponds and streams offer very different habitat conditions. Ponds for instance have a higher density of both, predators and conspecifics (Reinhardt et al., 2013; Thiesmeier & Mutz, 1997; Weitere et al., 2004). Both increase the predation pressure, as larvae can become cannibalistic in high densities. Because of these different habitat conditions, we hypothesise that (i) larvae from ponds show a more pronounced tonic immobility behaviour than larvae from streams; because of the higher predation pressure, and that (ii) bigger larvae display tonic immobility for a shorter period of time; as larger individuals are less likely to be predated on.

## 2 METHODS

We made the observations of the tonic immobility behaviour on April 4, 2023, at two pond and two stream locations while we were capturing larvae for another experiment. We started at 9:55 a.m. in the morning and visited the last location at 4:45 p.m. The ambient temperature varied between 10 and 14.7°C, while the water temperature varied between 4.4 and 8.1°C (mean ponds = 7.9°C, mean streams = 5.17°C).

The capturing and processing of the larvae was performed following Oswald et al. (2023). Briefly, after catching the larvae with a dip net, each larva was taken out of the dip net by carefully turning the dip net inside out into the water bucket to release the larva before further processing. The bucket was filled with water from the respective water body. After capturing all the larvae, we took the larvae individually out of the bucket with a dip net and released them carefully into the petri dish that was filled with tap water. We measured the total length by placing the petri dish on top of graph paper. Then, we transferred each larva carefully into the photo vessel by pouring the water and the larva slowly into the vessel for photographic identification. We always photograph the larvae from the right side of the body. However, both body sides can be used as independent sets for identification (Faul et al., 2022). The tap water that we used for the petri dish and the photo‐vessel was stored in the car already the day before the experiment which is why the temperature was similar to the ambient temperature. The tonic immobility behaviour of some of the larvae was induced when we transferred them from the petri dish into the small photo‐vessel by slowly pouring the water and the larva from one container to the other. Immediately after placing the larva into the vessel, the time (in seconds) was recorded during which the individual showed tonic immobility behaviour (see Video S1). We stopped the time when the individual completely turned around to the normal position, and we stopped the time after 120 s in cases where an individual showed the behaviour for more than 120 s (*N* = 1). We did not try to simulate a predator's attack by handling the larvae or inducing the tonic immobility behaviour on purpose or as a test (see, e.g., Passos et al., 2017), but observed the behaviour when it occurred, induced by human handling. Tonic immobility cannot only be provoked by a predator's threat but also by handling (e.g., Anaissi et al., 2020; Teles et al., 2017). All larvae were handled only by trained researchers.

Our aim was to understand if the larval habitat (pond or stream) affects tonic immobility. Therefore, we investigated first if the number of larvae showing tonic immobility differed between the two habitat types (pond or stream), by performing a Chi‐square test. Then, we investigated if the habitat influenced the duration of the behaviour. We used a linear mixed effect model with time as the dependent variable (response variable), habitat type (pond or stream) and size as fixed factors (predictor variables) and location (we sampled two locations per habitat) as a random factor. To find the best model fit, we used Akaike Information Criterion as well as the *performance* package (Lüdecke et al., 2021). We used the r package *lme4* (Bates et al., 2015). To calculate *R* ^2^ and semi‐partial *R* ^2^, we used the *partR2* package (Stoffel et al., 2021). All tests were carried out using R (version 4.3.2 (2023‐10‐31 ucrt)).

## 3 RESULTS

**FIGURE 1:**
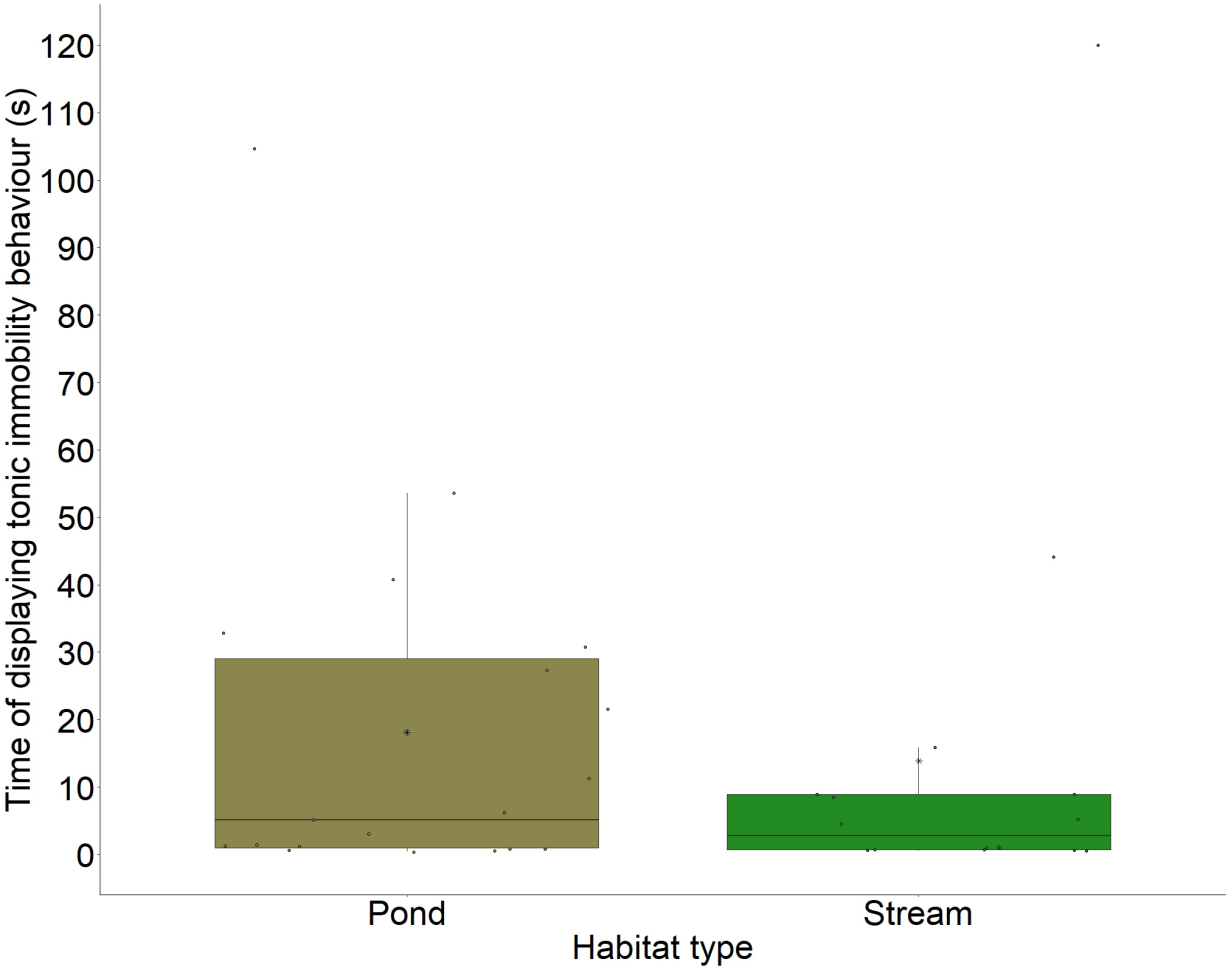
Time of displaying tonic immobility behaviour (s) for larvae from the two habitat types (pond and stream). Each point represents a single measurement. The horizontal line represents the median and the asterisks in the box represent the mean.

**FIGURE 2:**
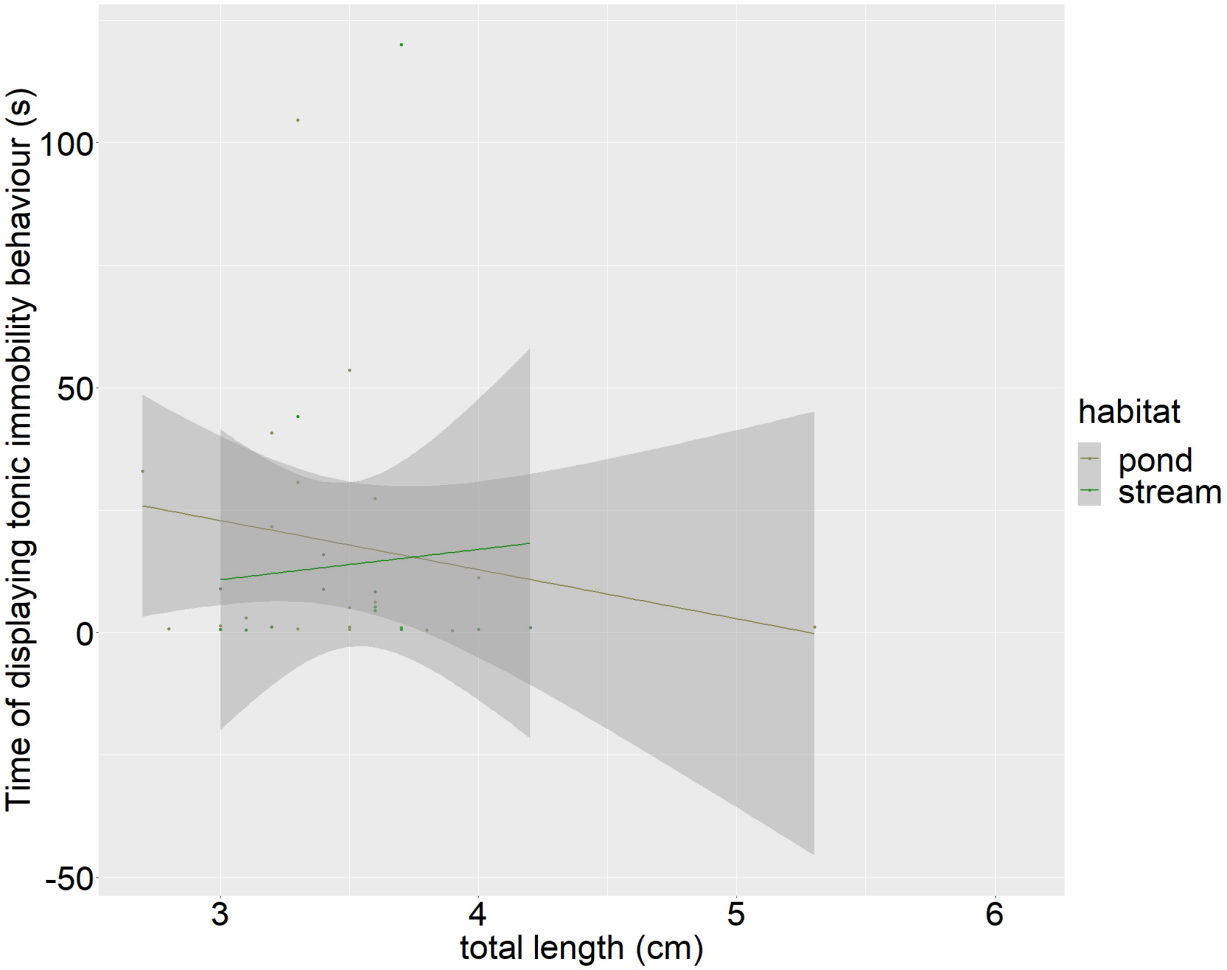
The total length (cm) of the larvae from both ponds and streams does not correlate with the time of displaying tonic immobility behaviour (s).

From the observed 221 larvae from both habitat types, a total of 35 larvae (about 16%) displayed the tonic immobility behaviour (Table 1). We found no significant difference in the proportion of larvae showing this behaviour between the two habitat types (Chi‐square‐test: χ^2^ = .166; *p* = .683). While 16 larvae showed this behaviour for 1 s or less, only two larvae showed it for more than 100 s (Figure 1). We did not find an effect of the size of the larvae (*p* = .376, Figure 2) and also not from the habitat type (pond or stream, *p* = .698, Table 2) on the tonic immobility behaviour. The marginal *R* ^2^ for our model was 4% while the semi‐partial *R* ^2^ for the habitat is 0%, for the total length 3.95% and for the combination of both predictors 4% (Table 3).

**TABLE 1:** Number of larvae observed and number of larvae that showed tonic immobility behaviour per habitat type (pond or stream).

	Number of larvae	Number of larvae showing tonic immobility behaviour
Pond larvae	130	19
Stream larvae	91	16
Total	221	35

**TABLE 2:** Linear mixed effect model (LMM) for the time the tonic immobility behaviour was displayed (s) regarding the habitat type (pond or stream) and the total length of the larvae.

Dependent variable	Fixed effect			
Time displaying tonic immobility (s)	Estimate	SE	*t* value	*p*
Intercept	1.232	1.323	0.932	.359
Habitat type	−0.186	0.404	−0.461	.698
Total length	−0.333	0.371	−0.898	.376

**TABLE 3:** Marginal *R* ^2^ and semi‐partial *R* ^2^ with lower and upper confidence interval (CI).

Predictor(s)	*R* ^2^	Lower CI	Upper CI
Model	.0404	0.0134	0.3085
Habitat type	0	0	0.2685
Total length	.0395	0.0126	0.3076
Habitat type + total length	.0404	0.0134	0.3085

## 4 DISCUSSION

Our study investigated potential differences in the tonic immobility behaviour from fire salamander larvae from two habitat types, ponds and streams. We also describe for the first time this behaviour in fire salamander larvae and that it can be induced by human handling.

Interestingly and against our hypothesis, we found neither an effect of habitat type, i.e., pond or stream, nor an effect of size on the time the tonic immobility behaviour was displayed by larvae. Likewise, the proportion of larvae from the two habitat types showing the behaviour did not differ.

One factor influencing tonic immobility is stress. Fijan ground frogs (*Platymantis vitiana*), for instance, showed longer TI behaviour when being exposed to a predator, which also led to an increase in stress hormones (Narayan et al., 2013). Thus, a relation between a more stressful habitat and a more pronounced tonic immobility behaviour was expected.

Recently, we have shown that fire salamander larvae from ponds and streams differ in their stress response (Schulte et al., 2023). As pond larvae showed higher baseline corticosterone responses, we expected larvae from ponds to show more likely and longer tonic immobility than larvae from streams. However, we did not find any difference between the two types, indicating that long term stress does not seem to influence the likelihood and the duration of tonic immobility.

Larvae from ponds and streams also differ in their risk‐taking behaviour. Oswald et al. (2020) showed that larvae from ponds are less risk‐prone, which is likely due to the higher predation pressure in their habitat. However, in our study, the tonic immobility behaviour was induced by the same threat, the human handling, which might have led to the same response in the fire salamander larvae irrespective of their natal habitat. It is unknown yet, in which other ways tonic immobility can be induced in fire salamander larvae. Thus handling is a first important step to study this behaviour, as handling is known to provoke a tonic immobility response in other species as well (e.g., Anaissi et al., 2020, Teles et al., 2017). Nevertheless, it does not represent a natural predator attack. Additionally, the larvae were placed in tap water, which did not contain any habitat specific kairomones and other chemical substances that predators release in the water (see Hahn et al., 2023). Thus, it can be assumed that the water did not contain any predator related information that could be perceived by the larvae.

Another factor that can influence the tonic immobility behaviour is satiation (Li et al., 2019). As the food supply differs quite substantially between pond and stream habitats, we expected a difference in the response. A possible explanation why the availability of food resources did not seem to influence the tonic immobility could be the time of the year. Our observations were made early in April, where densities of conspecifics are not too high, yet (see Oswald et al., 2023), and thus the food conditions can be assumed to be sufficient in ponds and streams.

Tonic immobility can be influenced by size and body weight. Hozumi and Miyatake (2005) found a correlation between body weight and the duration of tonic immobility behaviour in a beetle (*Callosobruchus chinensis*), with heavier beetles showing a longer tonic immobility behaviour. Likewise, fire salamander larvae from both ponds and streams were more risk‐prone when they were larger (Oswald et al., 2020). However, contrary to our expectations, we did not find an influence of body size. Antipredator defences usually come with a cost and thus lead to a trade‐off (Harvell, 1990; Mccollum & Van Buskirk, 1996). For the fire salamander larvae, the trade‐off might be different according to their habitat of origin. In streams, larvae showing tonic immobility can be seen well by a predator as the water usually is clear. However, the current of the stream can negatively affect the immobile larvae by drifting them further downstream into parts in which fish predators are present (Thiesmeier & Schuhmacher, 1990). This is particularly true for bigger larvae as the bigger body allows for a greater surface to be affected by the current. This does not apply to ponds as the water there is usually less clear and larvae cannot be seen as well. In this case however, the behaviour could be particularly advantageous as the density of predator is higher than in streams. Nevertheless, we did not find a difference in the tonic immobility behaviour that was displayed by pond and stream larvae despite the different habitat conditions and the potential different costs that might come with this.

In conclusion, we found that fire salamander larvae show tonic immobility, but we found no evidence for an effect of the larval habitat on this behaviour. Even though the tonic immobility was induced by handling, it probably does not reflect a natural predator attack. Future studies should consider mimicking a natural predator attack by one of the most common predators such as dragonfly larvae (in ponds and streams) and newts (in ponds) (Thiesmeier, 2004). This could for example be done by poking the tail with a stick or tweezers. Nevertheless, tonic immobility in fire salamander larvae is an interesting phenomenon that deserves further attention, especially considering that behaviour of fire salamanders is widely understudied (but see, e.g., Caspers et al., 2015; Hahn et al., 2023; Manenti et al., 2013; Oswald et al., 2020).

## AUTHOR CONTRIBUTIONS

**Laura Schulte:** Conceptualization (equal); data curation (lead); formal analysis (lead); methodology (equal); visualization (lead); writing – original draft (lead). **Barbara A. Caspers:** Conceptualization (equal); data curation (supporting); formal analysis (supporting); funding acquisition (lead); methodology (equal); project administration (lead); writing – review and editing (lead).

## FUNDING INFORMATION

This research was conducted within another experiment that was funded by the German Research Foundation (DFG) as part of the CRC TRR 212 (NC^3^) – Project numbers 316099922 and 396777092. Permissions were granted by the State Agency for Nature, Environment and Consumer Protection (LANUV; reference number: 81‐02.04.2021.A437), the nature reserve authority of the Stadt Bonn and the forest warden's office in Bonn. Experiments comply with the current laws of Germany. All larvae were released unharmed back into their pond or stream.

## CONFLICT OF INTEREST STATEMENT

We declare no conflict of interest.

## Supporting information


Video S1.

## Data Availability

Data and code can be found doi: 10.4119/unibi/2984984.
