# Supplementary material for: Tonic immobility behaviour does not differ between fire salamander larvae from ponds and streams
Source: Ecol Evol. 2024 Apr 1;14(4):e11211. doi: 10.1002/ece3.11211 (PMC10985366; doi:10.1002/ece3.11211)
Supplement: Supplementary file 1 — Video S1. [file ECE3-14-e11211-s001.zip › Video S1_legend.docx]

Video S1. Fire salamander larva (Salamandra salamandra) showing tonic immobility behaviour until returning to normal posture.
